# Clinical spectrum of female genital malformations in prenatal diagnosis

**DOI:** 10.1007/s00404-022-06441-3

**Published:** 2022-02-27

**Authors:** Michael R. Mallmann, Ulrich Gembruch

**Affiliations:** 1 Department of Obstetrics and Gynecology, University Hospital of Cologne, Kerpener Str. 34, 50924 Cologne, Germany; 2 Department of Obstetrics and Prenatal Medicine, University of Bonn, Bonn, Germany

**Keywords:** Female genital malformation, Fetus, Prenatal diagnosis

## Abstract

**Introduction:**

Fetal genital malformations represent a rare and heterogeneous group of congenital malformations of the disorders of sexual development (DSD) spectrum.

**Methods:**

A thorough literature review on the main topics in the prenatal approach towards DSD was conducted.

**Results:**

First, a thorough overview on prenatal characteristics of the most common fetal genital malformations of ovaries, uterus and external genitalia, and second, a standardized approach for differential diagnosis in the presence of direct and indirect prenatal signs of DSDs.

**Conclusions:**

This review is mainly directed towards the aspects of female genital malformations with  aspects of male DSD explained as well to aid in the prenatal differential diagnosis.

## Introduction

Fetal genital malformations represent a rare and heterogeneous group of congenital malformations of the disorders of sexual development (DSD) spectrum. In prenatal diagnosis, suspicion of DSD may arise in addition to abnormalities detected in other organ systems than the external and internal genitalia. Whereas in the postnatal setting extensive guidelines exist that focus on diagnostic and treatment and quantitative scoring systems such as the external masculinization score and the Prader score are commonly used for the description of externa genitalia, there exist only few standardized protocols for the ultrasound approach of fetuses with genital malformations [1–5]. With this manuscript we will give an overview on the main topics in the prenatal approach towards DSD: First, a thorough overview on prenatal characteristics of the most common fetal genital malformations of ovaries, uterus and external genitalia, and second, we propose a standardized approach for differential diagnosis in the presence of direct and indirect prenatal signs of DSDs. Although this review is mainly directed towards the aspects of female genital malformations, in the setting of external genitalia, aspects of male DSD are explained as well to aid in the prenatal differential diagnosis.

## Human female reproductive tract development

The female reproductive system consists of the gonads, their internal ductal system and the external genitalia. Embryologic development starts in the first trimester with the embryonic anlage [6].

The gonads are formed as genital ridges. The germ cells migrate into the gonadal anlagen in the 6th week and the primary germ cords are formed.

The development of the internal ductal system begins at the beginning of the 5th week, where the coelomic epithelium invaginates on the lateral surface of the urogenital ridges forming the Müllerian ducts that grow caudally within the urogenital ridges. In the cranial part of the urogenital ridge, Müllerian and Wolffian ducts are separated by mesenchyme, whereas more caudally, these two structures are separated only by the basement membrane or are even in direct contact. During the 7th and the 8th weeks, the right and left Müllerian ducts lie between the two Wolffian ducts. The two Wolffian ducts approach and join into the urogenital sinus, whereas the two Müllerian ducts fuse together during the 8th week and form a midline uterovaginal canal during the 9th week, as a temporary midline epithelium septum that separated the lumina of the two adjacent Müllerian ducts disappears. The uterine corpus develops from the cranial portion, the cervix from the middle two-fourths of the uterovaginal canal. The vaginal epithelium receives contributions from Müllerian duct and urogenital sinus that build the vaginal plate. The cavitation of this vaginal plate is started by the 16th week and almost completed by the 19th week. Mutations and aberrant methylation in genes involved in Müllerian and Wolffian duct development result in malformations of internal duct system [7].

The development of the external genitalia starts from the cloacal folds. These are formed in the 3rd week by a compression of the mesenchyme under the surface epithelium. In front of the cloacal membrane, the two folds unite and form the genital tubercle. With the division of the cloacal membrane into the urogenital membrane and the anal membrane in the 6th week, the cloacal folds are divided into the urethral folds and the anal folds. Until now, the genitalia are indifferent. By the influence of androgens, the male external genitalia develop. In female fetuses, the genital bulges on both sides of the urethral folds become the labia majora, the urethral folds the labia minora and the genital tubercle the clitoris.

## Ultrasound presentation of the fetal internal genitalia

### Fetal ovaries

**Fig. 1:**
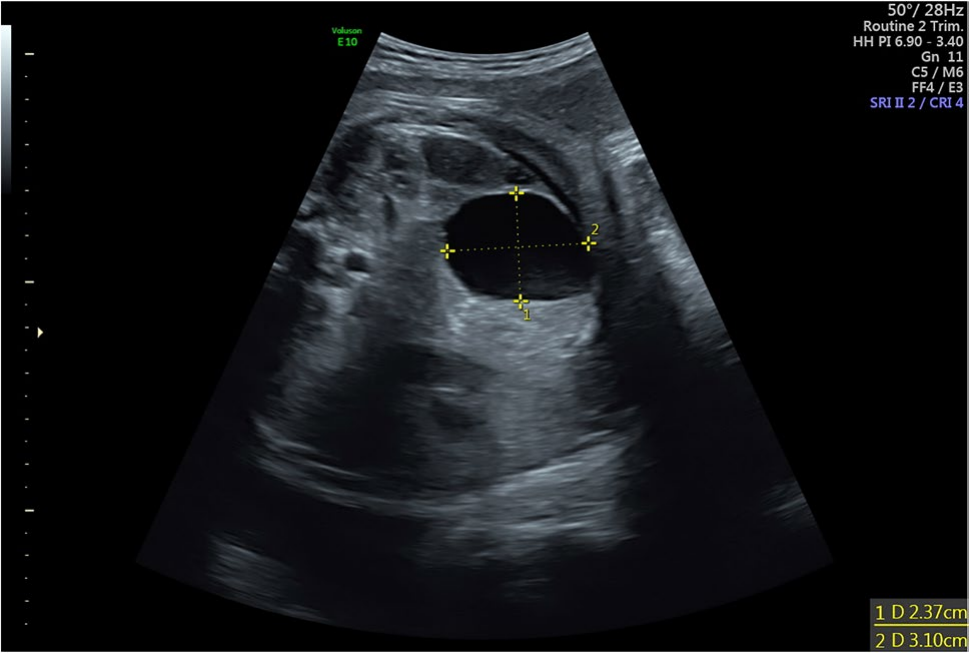
Ovarian cyst in a female fetus in 36 + 3 weeks of gestation

Due to their size, unsuspicious fetal ovaries cannot be visualized during fetal development. Consequently, the most commonly diagnosed ovarian anomalies are associated with a substantial ovarian enlargement. Stimulation of fetal ovaries by fetal follicle-stimulating hormone (FSH), maternal estrogen or placental human chorion gonadotropin (HCG) result in the occurrence of fetal ovarian cysts (Fig. 1). In general, ovarian cysts are categorized into (1) simple, anechoic, unilocular and thin-walled cysts and (2) complex, thick-walled cysts containing intracystic septations and hyperechogenic parts, resulting from ovarian torsion or intracystic hemorrhage [8]. Some cysts show spontaneous regression during pregnancy, whereas most cysts will only regress after birth. The risk of torsion of the ovary increases with the size of the cyst and is increased 30-fold in cysts measuring ≥ 40 mm than in cysts < 40 mm [8]. Intrauterine puncture of simple cysts with a size of ≥ 40 mm during pregnancy might be performed to lower the risk of intrauterine ovarian torsion, yet in 40%, the cyst will reappear due to the still existing hormonal stimulus [8, 9].

### Fetal uterus and vagina

In many female genital abnormalities, visualization of the fetal uterus is relevant for the classification of the abnormality, especially in the case of ambiguous external genitalia as discussed below. We suggest an attempt to visualize the fetal uterus whenever genital, renal or urinary anomalies are detected. The fetal uterus can be identified with 2D-ultrasonography starting with the 19th week of gestation and shows a linear growth throughout pregnancy [10]. However, in 20% of female fetuses, the uterus cannot be detected with this technique. With the use of three-dimensional volume contrast imaging, detection rates are less than 50% of cases at 20–22 weeks of gestation, but ~ 80%% of cases at 32–34 weeks of gestation [11]. If direct visualization of the fetal uterus is not possible, indirect ultrasound signs exist that might ease in the diagnosis. Glanc et al. determined the fetal sex correctly in 98.8% of female fetuses and 100% of male fetuses between 14 and 40 weeks of gestation using the fact that the presence of the uterus in female fetuses results in an increase in the distance between bladder and rectum as compared to male fetuses and the concave indentation of the posterior aspect of the bladder by the uterus [12].

Malformations of the fetal uterus and/or vagina mainly arise from Müllerian agenesis, an embryologic underdevelopment of the Müllerian duct resulting in agenesis or atresia of the uterus, vagina, or both, commonly referred to as Mayer–Rokitansky–Küster–Hauser (MRKH) syndrome. Müllerian duct agenesis might be diagnosed during puberty in patients evaluated for primary amenorrhea, but prenatal diagnosis depends on the extend of Müllerian duct agenesis. As MRKH syndrome is characterized by the aplasia of uterus and the upper part of the vagina, female fetuses with MRKH are typically missed in prenatal diagnosis due to missing ultrasound signs [13].

**Fig. 2:**
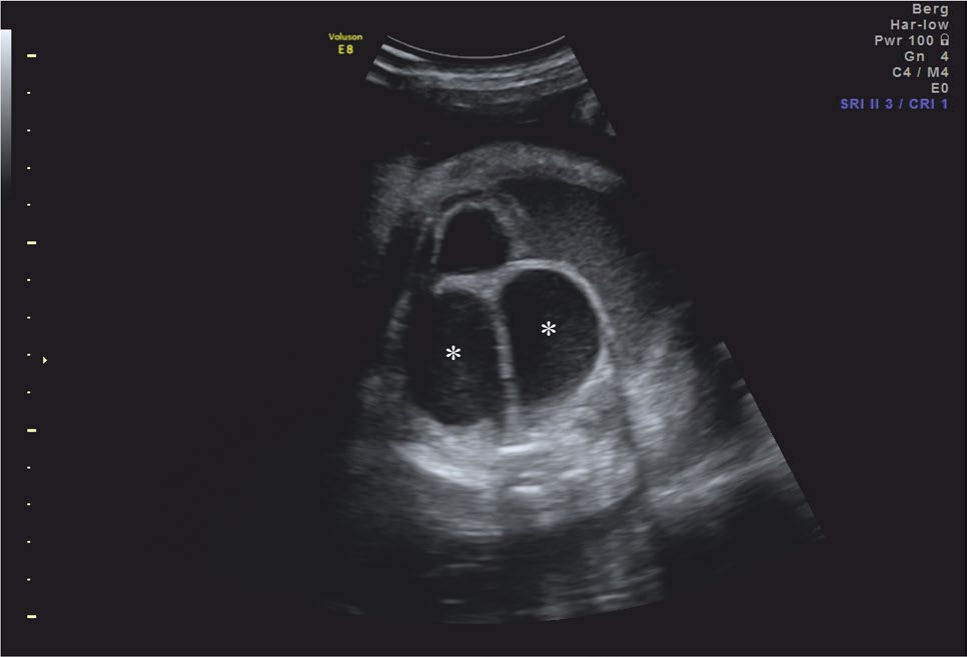
Hydrometrocolpos in a female fetus in 33 + 2 weeks of gestation. A fluid-filled vagina duplex (*) with sludge phenomenon connected to a fluid-filled uterus

Failure of regression of the septum results in an imperforate hymen, the most common reason for hydrometrocolpos. Hydrometrocolpos represents a fluid accumulation in the upper vagina and uterus that typically appears in late pregnancy [14]. In ultrasound, hydrometrocolpos often presents as a retrovesical, septate hypoechogenic mass within the lower fetal abdomen (Fig. 2) [14].

Differential diagnosis of intraabdominal cystic masses most often is difficult and includes hydronephrosis, fetal ovarian cysts, anterior cystic teratoma, OEIS (Omphalocele–Exstrophy–Imperforate anus–Spinal defects) complex, megacystis with lower urinary tract obstruction, hepatic cysts, fetal sacrococcygeal teratoma, anterior meningocele and hydrometrocolpos [14]. Dependent on the cause, four types can be distinguished: type 1 due to an imperforate hymen, type 2 due to a transverse septum of the vagina, type 3 due to vaginal atresia and type 4 due to vaginal atresia and persistence of urogenital sinus [15].

Hydrometrocolpos may be isolated, but is often part of a syndromic disease or result of a spectrum of urogenital malformations: Hydrometrocolpos is most commonly associated with urogenital malformations that originate from embryological failure of urethrovaginal and/or anorectal septation and result in cloacal malformations [14]. Consequently, cloacal malformation should be ruled out first in the appearance of hydrometrocolpos. Prenatal presentation of hydrometrocolpos with polydactyly is mainly related to the McKusick–Kaufman (MKKS) and Bardet–Biedl (BBS) syndromes. MKKS is characterized by the combination of polydactyly, hydrometrocolpos and heart defects (15–20%). In contrast, BBS is characterized by the combination of polydactyly and a urogenital malformation (hydrometrocolpos being the most common urogenital malformation in BBS). Renal malformation is often, but additional pathognomonic features such as obesity and retinitis pigmentosa only present later in life. Due to the substantial overlay in both syndromes an exact diagnosis in pregnancy remains difficult and consequently, these fetuses are prenatally often classified as being part of the MKKS/BBS spectrum [14, 16].

Female fetuses with MURCS (MUllerian agenesis, Renal agenesis, Cervical thoracic Somite abnormalities) association present with hydrometrocolpos as well as do fetuses with features of a VACTERL (Vertebral, Anal, Cardiac, Trachea–Esophageal fistula, Renal/kidney, Limb defects) association [14, 17]. Type II MRKH is associated with additional malformations generally affecting the renal and skeletal systems, in which the MURCS association is specially characterized by cervico-thoracic defects [13, 18, 19].

Pallister–Hall syndrome should be ruled out if hydrometrocolpos is associated with intrauterine growth retardation, short extremities, hexadactyly and genital malformation. Intracerebral hamartoma is pathognomonic for this syndrome and cMRT should be carried out to exclude this syndrome in children [14].

A combination of hydrometrocolpos with unilateral renal anomaly (agenesis or dysplasia) is typical for Herlyn–Werner–Wunderlich syndrome (HWWS) or OHVIRA syndrome (obstructed hemivagina and ipsilateral renal anomaly), a rare anomaly characterized by Müllerian duct anomalies associated with mesonephric duct anomalies [14, 20].

In female fetuses with a combination of genital tract malformations, especially vaginal aplasia or a rudimentary or bicornuate uterus, and renal cysts, the Renal cysts and diabetes syndrome should be considered. This syndrome includes prenatal ultrasound findings of the kidneys (agenesis, hypoplasia, cysts; unilateral or bilateral pattern), genital tract and pancreas [21]. Mutations of hepatocyte nuclear factor-1 (*HNF1B* gene) will confirm fetuses with this syndrome.

## Ultrasound presentation of the fetal external genitalia

**Fig. 3:**
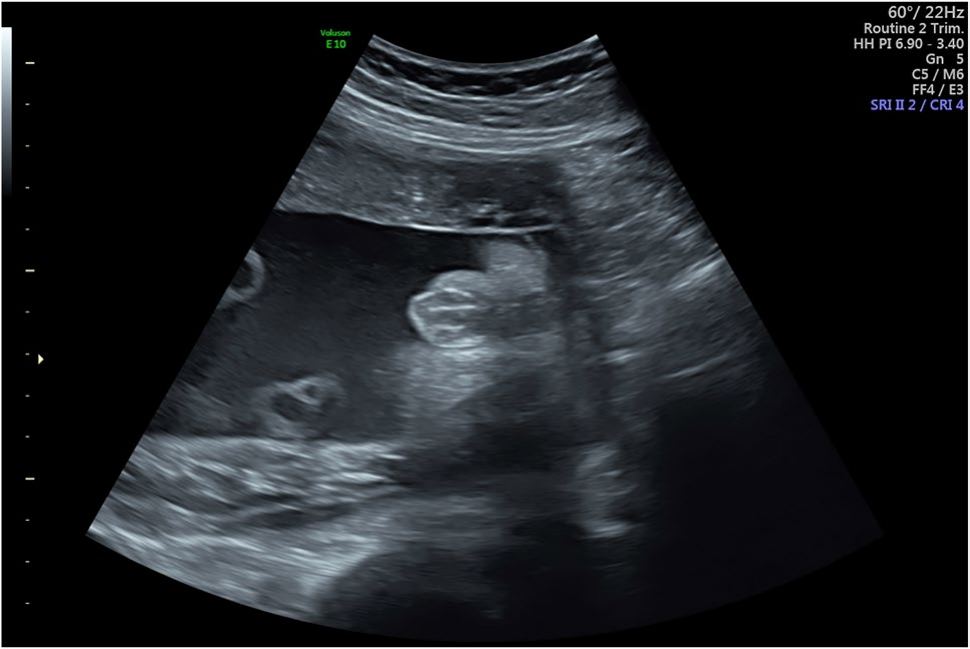
Fetal labia in 30 + 4 weeks of gestation. Characteristic multiple parallel linear echo pattern in a female fetus

**Fig. 4:**
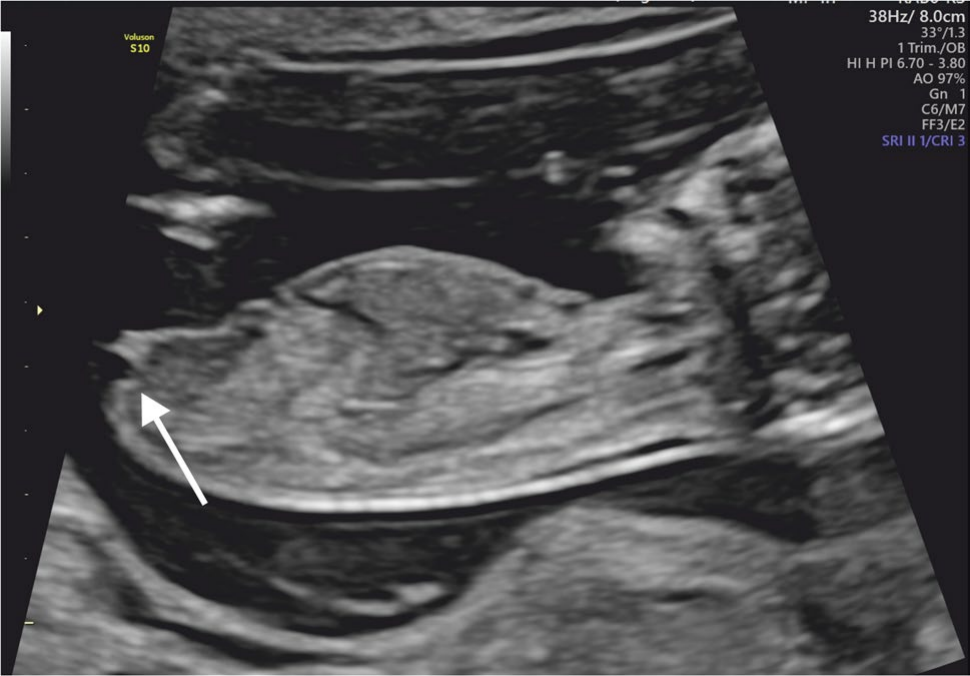
Female fetus in 13 + 0 weeks of gestation. Genital tubercle parallel to the horizontal line of the fetus (arrow) suggesting female gender in a fetus in the first trimester

**Fig. 5.**
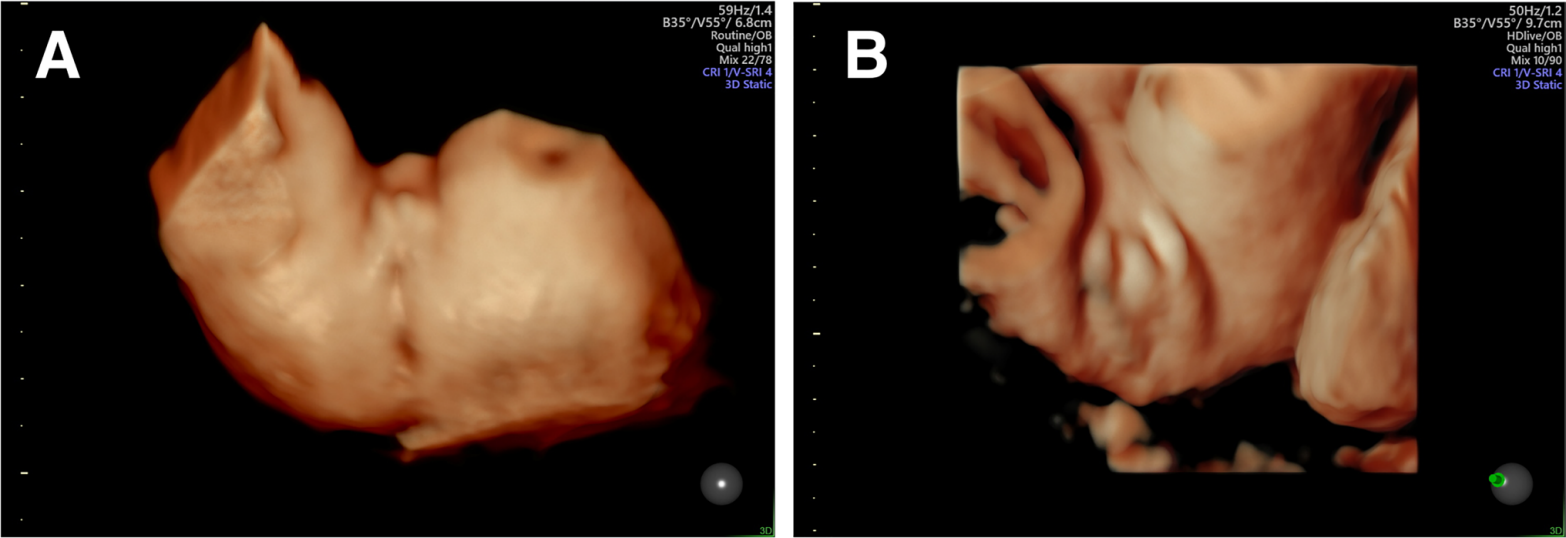
3D sonography of the external female fetal genitalia in **a** 21 + 0 and **b** 32 + 2 weeks of gestation

Ultrasonographic evaluation of the fetal external genitalia plays a pivotal role in the evaluation of normal fetal sex development. Incorrect determination of fetal sex has severe implications affecting the mental and psychological health of the mother and the upbringing of the newborn [22]. Fetal sex determination has been described as early as 1977 by Stocker and Evens, who examined the fetal perineal area after 30 weeks of gestation and described an overall success rate of > 95.6% with this technique (correct in 99.5% of those diagnosed as males and in 91.5% of those diagnosed as females) [23]. With the improvement in ultrasound technique, fetal sex determination shifted towards novel ultrasound signs and earlier detection. By 15 weeks of gestation, the female labia may be visualized as a multiple parallel linear echo pattern with the midline cleft separating the labial folds (Fig. 3) [24]. Between 10 and 14 weeks of gestation, fetal gender can be predicted by the assessment of the direction of the genital tubercle points, cranial for males and caudal for females, as there is no difference in the size of the fetal clitoris and the fetal penis until 14 weeks of gestation. The sagittal sign introduced by Emerson et al. is a valid ultrasound sign of fetal sex with improving accuracy between 10 and 20 weeks of gestation [25]. Within a midline sagittal view of the caudal end of the fetal torso, the contour of the rump is followed from dorsal to ventral until a focal bulge with an angular notch at the cranial or caudal edge on it is found. A caudal notch indicates female, a cranial notch indicates male genitalia. This ultrasound sign has been shown to be most accurate between 14 and 20 weeks of gestation with an correct gender assignment of 75% between 12 and 14 weeks of gestation and nearly 100% after 14 + 0 weeks of gestation [25]. Efrat and Nicolaides report on a technique using the angle of the genital tubercle (Fig. 4) [26]. The fetal gender is assigned as male if the angle is greater than 30° and female if the phallus is parallel or convergent (less than 30°) to the horizontal line. The accuracy of this technique increases with fetal crown–rump length and gestational age, has a success rate between 70% at 11 weeks and 100% at 13 weeks and its use has been validated [26–28]. With the introduction of the anogenital distance (AGD), Arfi et al. added another powerful ultrasound sign for the determination of fetal sex between 11 and 13 + 6 week of gestation [29]. As the distance between the caudal extremity of the fetus and the base of the genital tubercule is testosterone dependent, measurement of the AGD demonstrated a high accuracy in distinguishing male from female fetuses at a cut-off of 4.8 mm resulting in correct sex determination in 87% of the males and 89% of the females. Finally, Kurban et al. found that male and female show a significant difference in their yolk sac-fetal pole distance [30]. With this technique, the distance between the fetal pole and the yolk sac is measured in millimeters (mm), when the fetal pole is in the longitudinal position. With a cut-off of 1.8 mm, a 70% sensitivity and 67% specificity for female gender prediction is reached. In addition to the aforementioned 2D measurements, also 3D-ultrasonography has been studied in their predictive capability of sex determination (Fig. 5). Although it might be a useful tool in conjunction with traditional 2D-ultrasonography for certain questions, its accuracy in sex determination has been shown to be not superior to 2D- ultrasonography in both first and second trimester [31, 32].

### Fetal anomalies of the external genitalia/ ambiguous genitalia

Malformations of the external genitalia are subsumed under the term “disorder of sex development (DSD)” as recommended by the Chicago consensus statement [33]. In this nomenclature, aberrations of the sex chromosome (“Sex chromosome DSD”) are distinguished from DSD without aberrations of the sex chromosome (46,XX DSD and 46,XY DSD). Prenatal issues with respect to DSD generally arise with (a) a discrepancy between genetic sex as determined by amniocentesis or cell-free fetal DNA and the phenotypic sex as determined by ultrasonography or (b) fetal ambiguous genitalia on ultrasound.

The ultrasound diagnosis of fetal ambiguous genitalia (prevalence ~ 1:5000 births) may be suspected in the case of incomplete masculinization of the external genitalia (abnormal shape, absent or short phallic structure and/or an absent or bifid scrotum with absent or undescended testes in late pregnancy and a short recto-vesical space), a masculinization of the female external genitalia (enlarged phallic structure and abnormal/fused labia instead of scrotum, an identifiable uterus or enlarged recto-vesical space) or a discordance between external and internal fetal gender [34].

## Sonographic characteristics of fetuses with DSD according to the type of disorder

### Sex chromosome DSD

#### 47,XXY (Klinefelter syndrome and variants)

Klinefelter syndrome (47,XXY) is one of the most common sex chromosome disorders with an incidence of 1/500 to 1/800 male fetuses. Only 10% of cases are diagnosed prenatally, as its symptoms prenatally are mild, if there are any detectable and most cases are never detected [35]. Nowadays, most fetal cases are detected accidentally by NIPT screening. In ultrasound, non-specific symptoms such as an increased nuchal translucency might result in non-directed genetic testing. With increase in severity of polysomy in the Klinefelter variants 48,XXXY and 49,XXXXY, more severe clinical abnormalities may be detected prenatally: cystic hygroma in the first trimester and micropenis, clubfoot and polyhydramnios in the second trimester [36].

#### 45,X (Turner syndrome and variants)

Turner syndrome (45,X) is the most common chromosomal abnormality in females with an incidence of 1 in 2500 female livebirths. The majority of fetuses has a 45,X genotype, one third 45,X mosaicism (45,X/46,XX) and the rest isochromosome X (isochromosome Xq), mosaicism 45,X/46,XY (mixed gonadal dysgenesis) or ring X karyotype. The most common ultrasound sign in early pregnancy remains large cystic hygroma and hydrops fetalis, which are somehow pathognomonic for Turner syndrome. A fetal heart rate above the 95th percentile, sonographic signs of pleural effusion, an abnormal ductus venosus velocimetry and a right dominant heart should also rise suspicion for fetal Turner syndrome [37]. Even in fetuses with fetal megacystis, a substantial proportion is diagnosed with Turner syndrome [38]. In later pregnancy, structural heart defects such as coarctation of the aorta and hypoplastic left heart might be detected. Turner syndrome is associated with gonadal dysgenesis, yet this feature is undetectable in utero and genitalia of fetuses with Turner syndrome are of normal female morphology. Most fetuses with Turner syndrome will die in utero and only 1% will survive until term.

#### 45X/46XY (mixed gonadal dysgenesis)

45X/46XY mosaicism results from a mitotic error in a single zygote. The cytogenetic finding of 46,XX and 46,XY cells at prenatal genetic is reported at a rate of 1.5 in 1000, the vast majority of cases being the result of contamination of normal fetal male cells with maternal cells. Consequently, this technical aspect must be ruled out in each case of a 45X/46XY mixed gonadal dysgenesis genotype. If mixed gonadal dysgenesis is assured, phenotype depends on the extent of mosaicism in the gonads and other affected tissues. The majority of fetuses (80%) with a 45X/46XY genotype will present as phenotypic males with normal male genitalia [39]. Nevertheless, a substantial proportion will present with the following genital abnormalities: hypospadia, micropenis, an abnormal scrotum, unilateral cryptorchidism and scrotal hydrocele in phenotypic males, clitoromegaly or a rudimentary uterus in phenotypic females. These fetuses may also present as true hermaphrodites with abnormal internal genitalia with testes on one and ovary and uterus on the other side [40]. Extragenital anomalies include meningomyelocele, malformations of ears, hands and feet and cardiac malformations such as small ventricular defects [39]. Chang et al. describe a cohort of 92 pregnancies with fetuses with a 45X/46XY genotype resulting in 40% termination of pregnancy (TOP), 5% demise during pregnancy and 50% live births [39]. Fetuses with a 45,X/46,X,idicY karyotype represent a variant, which, similar to fetuses with a 45X/46XY genotype will present with normal male rather than female phenotype in the majority of cases (75%) [41].

#### 46XX/46XY (chimerism)

45X/46XY chimerism results from the fusion of two different zygotes in a single embryo. Its incidence is extremely low and only single case reports on the prenatal presentation have been published. Most cases are described as fetuses with (a) normal female external genitalia, a complete uterus and gonads or (b) normal male external genitalia, all with a successful pregnancy outcome [42–44]. Nevertheless 45X/46XY chimerism warrants detailed ultrasound of external and internal fetal genitalia as it might also present with true hermaphrodites with an normal male external genitalia and testes on one yet ovary and uterus on the other side [42].

#### 47,XXX (Triple X syndrome)

Triple X syndrome has an incidence of 1:1000 female births. Triple X is most commonly diagnosed prenatally randomly by means of an NIPT test result. Only in some cases, genetic testing is performed after the diagnosis of minor abnormalities such as ventriculoseptal defects, clubfoot or singular umbilical artery [45]. External and internal genitalia are usually not altered in prenatal ultrasound.

### 46,XY DSD

#### Disorders in androgen synthesis

Missing stimulation by mutation in the luteinizing hormone receptor and the inability to transform testosterone to 5-dihydrotestosterone, i.e., 5α-reductase deficiency and sometimes 17β-hydroxysteroid dehydrogenase deficiency, may lead in genetic males to the formation of female genitalia.

Whereas cases with mutation in the luteinizing hormone receptor have been described postnatally, to our knowledge, none have been reported in the prenatal setting.

17β-hydroxysteroid dehydrogenase deficiency and 5α-reductase deficiency are rare autosomal-recessive inherited conditions. 17β-hydroxysteroid dehydrogenase converts androstenedione to testosterone and 5α-reductase converts testosterone to the more potent 5α-dihydrotestonsterone. The clinical spectrum is heterogeneous at birth, ranging from a female with a blind vaginal pouch to a fully male phenotype with hypospadias and micropenis. Despite introduction of NIPT, prenatal diagnosis is uncommon in sporadic cases. Genetic diagnosis can be made by amniotic fluid analysis.

#### Disorders in androgen action

Disorders in androgen action arise with mutation in the androgen receptor that result in the Androgen insensitivity syndrome or endocrine disruptors that interact with androgen action.

Androgen insensitivity syndrome (AIS) is an X-linked recessive genetic disorder caused by mutation in the androgen receptor. It is characterized by the presence of a female phenotype in the presence of bilateral testes and an XY karyotype. Due to a several hundred different mutations been reported to results in androgen insensitivity, the clinical spectrum is wide and can be divided into three major external genital phenotypes: complete androgen insensitivity with a normal or near-normal female phenotype with testes being either abdominal or descended; partial androgen insensitivity with an ambiguous phenotype, a phallic urethra or penoscrotal hypospadias and undescended testes and mild androgen insensitivity with a nearly normal male phenotype yet small penis and scrotum, sometimes coronal hypospadias and descended testes [46, 47]. Only few cases have been described in the prenatal setting. Most often, the condition is diagnosed prenatally in the setting of a discrepancy between male karyotype, female external genitalia yet missing female internal genitalia (no uterus visible) [46–49].

Endocrine disruptors constitute an extremely heterogenous group of substances that fetuses can be exposed to during pregnancy and that potentially affect different endocrine systems. If we consider only the estrogen and testosterone system, several endocrine disruptors have been associated with altered fetal genitalia and most of these substances have antiandrogenic and/or mild estrogenic effects and mainly cause ambiguous genitalia in male infants [50]. Among these substances are phthalates, polychlorinated biphenyl (PCB), dichlorodiphenyltrichloroethane (DDT) and many more [50, 51]. Endocrine disruptors should be included in differential diagnoses especially in fetuses with intrauterine growth retardation combined with 46,XY DSD such as hypospadias and undescended testicle.

### 46,XX DSD

#### *Testicular DSD (SRY* +*)*

The 46,XX male syndrome is an extremely rare genetic disorder that is found in 1:25,000. The phenotype depends on the varying amounts of Y material including the Y boundary and SRY and can be classified into three groups: (1) classic XX males with normal male internal and external genitalia, (2) XX males with ambiguous genitalia such as hypospadias, micropenis, or clitoromegaly and (3) XX true hermaphrodites presenting with internal or external genital ambiguities [52].

#### Androgen excess

Timing of androgen excess during pregnancy results in a different amount of virilization of the female fetal genitalia. Androgen exposure from 8th through 12th week of gestation results in both fusion of the labioscrotal folds and clitoromegaly, whereas androgen exposure exclusively after the 12th week of gestation rather results in singular clitoromegaly. Only few disorders of steroid metabolism are associated with androgen excess in the prenatal setting, mainly fetal congenital adrenal hyperplasia and the rare fetoplacental aromatase deficiency [53].

##### Fetal: congenital adrenal hyperplasia (CAH)

Congenital adrenal hyperplasia is caused by deficiencies of one of the enzymes required for the synthesis of cortisol in the adrenal glands. The result is an accumulation of mineralocorticoid precursors and an increased production of adrenal androgens. More than 90% of CAH are caused by mutations in the 21-hydroxylase gene, 5–8% by mutations in the 11β-hydroxylase gene and some by mutations in the 3 beta-hydroxysteroid dehydrogenase gene. It has an incidence of 1:15.000 births. In the postnatal setting, 70% 70% of affected children present with a combination of salt-wasting and virilization, 30% show a simple virilization. In the prenatal setting, female fetuses develop ambiguous genitalia.

##### Fetoplacental: aromatase deficiency

Aromatase deficiency results from autosomal-recessive inheritance of mutations in the *CYP19A1* gene encoding the enzyme aromatase. The placenta aromatizes large quantities of androgens into estrogen by use of the aromatase enzyme. As the placenta represents fetal tissue fetal mutations in the *CYP19A1* gene result in massive increase in androgen levels both affecting mother and fetus. In the mother, the high amounts of testosterone that are equivalent to that of males result in the development of cystic acne, hirsutism and clitoromegaly [54]. In contrast to virilizing maternal ovarian tumors, maternal ingestion of androgens or androgenic drugs, where high level of androgens are found together with normal levels of estrogen in the maternal serum, aromatase deficiency is characterized by extreme low levels (0.1–8% of normal values) of estradiol and estriol levels in the maternal serum [55]. 46,XX fetuses present with ambiguous genitalia with severe clitoromegaly and posterior labioscrotal fusion [55].

##### Maternal (virilizing tumors or androgenic drugs during pregnancy)

Maternal virilizing tumors represent extreme rare causes of virilization of female fetuses. Mothers typically present with signs of virilization such as hirsutism and acne. Maternal adrenocortical carcinoma, maternal Ovarian Luteoma and maternal Krukenberg tumors have been associated with this condition [56–58]. The 46,XX fetuses present with ambiguous genitalia with clitoromegaly and fusion of the labioscrotal folds.

Maternal use of drugs structurally related to androgens might result in the virilization of external fetal female genitalia. The most relevant medication that might result in virilization of female fetuses nowadays is the androgen danazol, a medication used to treat severe endometriosis.

## Syndromes and associations that may include DSD

Prenatal diagnosis of DSD is extreme rare and may raise diagnostic and prognostic dilemma in the prenatal setting. In the presence of additional malformations and syndromic fetuses, overlapping conditions makes accurate diagnosis challenging (Table 1). Moreover, the exact criteria for diagnosis, in terms of the number and nature of anomalies included, are controversial in most syndromes.

**Table 1:** Genetic syndromes associated with prenatal genital malformations

Syndrome	Genetic background	Fetal genitalia	Presentation in prenatal ultrasound	References
*Trisomy 18*	Trisomy 18	Rarely ambiguous genitalia	Nuchal translucency, growth retardation, choroid plexus cysts, overlapping of fingers and congenital heart defects	
*Cornelia de Lange syndrome*	Several genes	46,XY DSD: hypospadias, undescended testes	Moderate to severe growth retardation + distinct upper limb abnormalities (agenesis, brachydactyly, oligodactyly or shortening of the hands) + facial abnormalities (long, smooth philtrum alone or together with an upturned, small nose and retrognathia, long eyelashes)	[74, 75]
*Fraser syndrome (cryptophthalmos–syndactyly syndrome)*	Mutations in FRAS1, GRIP1, FREM2 genes	Ambiguous genitalia with micropenis or clitoromegaly	Diagnosis made in the presence of two major and one minor or in the presence of one major and at least four minor criteria: Major diagnostic criteria (cryptophthalmos, syndactyly, abnormal genitalia, a sibling with Faser syndrome) Minor diagnostic criteria (malformation of the nose, malformation of the ear, malformation of the larynx, renal agenesis, clefting, skeletal defects and umbilical hernia)	[76, 77]
*Frasier syndrome* * Type 1* * Type 2* * Type 3*	Mutations in in intron 9 of the *WT1* gene Karyotype 46,XY Karyotype 46,XY Karyotype 46,XX	46,XY DSD, 46,XX DSD	46,XY DSD: external genitalia range from normal female external genitalia to ambiguous genitalia with penoscrotal hypospadias or microphallus; the uterus can be fully developed or absent; the gonads can present as normal testes as well as small or streak gonads 46,XX DSD: normal female genitalia; the uterus might be normal or bicornuate; the gonads show streak gonads or dysgenetic testes	[78–80]
*WAGR (Wilms tumor-aniridia) syndrome*	Microdeletion of 11p13 including the *WT1* and the *PAX6* gene	46,XY DSD		
*Denys–Drash-syndrome*	Mutations in introns 8 or 9 of the *WT1* gene	46,XY DSD		
*Fryns syndrome*	Mutations in the PIGN gene	46,XY DSD: ambiguous external genitalia with appearance of bifid scrotum versus thickened labial swellings and no identifiable phallus 46,XX DSD: bicornuate uterus	Diaphragmatic hernia + characteristic “coarse” facies, clefting, distal digital hypoplasia, polyhydramnios, brain malformations (hydrocephalus, abnormalities of the corpus callosum, Dandy–Walker malformation), cardiovascular malformations, renal dysplasia	
*Pallister–Killian syndrome*	Mosaic tetrasomy 12p + isochromosome 12p	46, XY: hypoplastic genitalia 46,XX DSD: normal genitalia	Diaphragmatic hernia, polyhydramnios, rhizomelic micromelia, fetal overgrowth, hydrops fetalis, hygroma colli, brain abnormalities	[81]
*Noonan syndrome*	Mutations in genes of the RAS-MAPK pathway	46, XY: cryptorchidism or monarchism 46,XX DSD: normal genitalia	Large variety with cystic hygroma, increased nuchal translucency, hydrops, polyhydramnios, lymphatic dysplasia, pleural + pericardial effusions, ascites, renal anomalies, cardiac anomalies	[82]
*Robinow syndrome*	Mutations in the *ROR2* gene	46, XY: scrotum without penis/micropenis 46, XX: clitoral hypoplasia	Mesomelic limb shortening, facial malformations	[83–85]
*Silver–Russell-Syndrome*	Genetically heterogenous	46, XY: cryptorchidism 46, XX: congenital aplasia/hypoplasia/dysplasia of the uterus, ambiguous genitalia with clitoral hypertrophy, absent ovaries, periscrotal hypospadias	Severe growth retardation with relative macrocephaly, triangular face with micrognathia and/or low-set and abnormal ears, limb asymmetry, fifth finger clino-/brachydactyly	[86–88]
*Smith–Lemli–Opitz syndrome (SLOS)*	Mutations in the *DHCR7 gene*	46, XY: ambiguous genitalia with micropenis, hypospadias or cryptorchidism 46, XX: normal genitalia	First trimester: increased nuchal translucency Second/third trimester: IUGR, malformations of extremities (postaxial polydactyly, syndactyly of toes 2–3, brachymelia, brachydactylia), the palate (cleft palate), brain (corpus callosum agenesis, hypoplastic cerebellum, complete or incomplete agenesis of the vermis, holoprosencephaly), microcephaly, facies (bitemporal narrowing, short nose, anteverted nostrils, long philtrum, microgenia, low-set ears), heart (septal defects) and kidneys (renal hypoplasia or unilateral renal agenesis)	[89–92]
*CHARGE association*	Mutations in the *CHD7 gene*	46, XY: genital hypoplasia with micropenis, penile agenesis, hypospadias or undescended testis 46, XX: genital hypoplasia with hypoplastic labia majora, minora and clitoris and atresia of uterus, cervix and vagina	Existence of four major criteria or the existence of three major and three minor criteria: Four major criteria: ocular coloboma, choanal atresia, ear abnormalities and cranial nerve involvement Seven minor criteria: genital abnormalities, development delay, cardiovascular malformations, growth retardation, orofacial cleft, tracheoesophageal fistula, minor facial abnormalities	[93–97]
*Camptomelic dysplasia*	Mutations in the SOX9 *gene*	Ambiguous genitalia, with discordance of internal and external genitalia, discordance between male genotype and ambiguous or even female fetal genitalia	Bowed tibiae, hypoplastic scapulae, flat facies and shortened, poorly ossified long bones	[98]
*Simpson–Golabi–Behmel syndrome*	Mutations in the CPC3 *gene*	ambiguous genitalia	Overgrowth syndrome: macrocephaly, macrostomia, macroglossia, large hands with postaxial polydactyly, clefting and/or coarse facial features	

## Malformations of the anorectal, genito-urinary and sacral anomalies (ARGUS) spectrum

### VATER/VACTERL association

The acronym VATER/VACTERL association refers to the rare, non-random co-occurrence of the following component features (CFs): vertebral defects (V), anorectal malformations (ARM) (A), cardiac defects (C), tracheoesophageal fistula with or without esophageal atresia (TE), renal malformations (R), and limb defects (L) [59]. The diagnosis is made in the presence of at least three CFs [60]. Genital anomalies are not classically associated with VACTERL association and some even argue the existence of external and internal genital anomalies to be unique for the urorectal septum malformation sequence (URSM) sequence in contrast to VACTERL [61]. Malformations of the external genitalia have rarely been described in several cases and include bifid scrotum, a caudally displaced, dysplastic penis and hypospadias [62].

### Partial URSMS (urorectal septum malformation sequence)

Urorectal septum malformation sequence (URSMS) is characterized by urethral obstruction, imperforate anus, ambiguous genitalia, renal agenesis or dysplasia, and mullerian duct maldevelopment. The complete URSMS shows an incomplete partition of cloaca with incomplete canalization of cloacal membrane, no perineal opening and is usually lethal. The partial URSMS shows an incomplete partition of cloaca with rupture of cloaca membrane, resulting in one perineal opening. The URSMS are associated with ambiguous genitalia and urogenital, colonic, and lumbosacral anomalies. In fetuses with partial URSM, malformations of both internal and external genital have been regularly reported. In females, the external genitalia malformations consist of a cloaca with a single opening, fusion of the labia and the existence of phallic like structures, the internal genitalia malformations of the unilateral existence of ovaries, the existence of a bifid vagina and the existence of a bicornuate or bifid uterus [61]. In males, the existence of a cloaca with a single opening, a bifid scrotum, hypospadias, penoscrotal transposition and penial aplasia and dysplasia as well as cryptorchidism have been reported [61].

### Bladder exstrophy–epispadias complex (BEEC)/cloacal malformations

The BEEC represents a spectrum ranging from isolated epispadias over isolated bladder exstrophy to the most severe form, cloacal exstrophy. Cloacal malformations represent the most severe form of the exstrophy–epispadias complex and might be accompanied by omphalocele, lumbosacral spine malformations such as hemivertebrae, segmentation defects and menigomyeloceles and ambiguous genitalia, the often referred to as OEIS (Omphalocele–Exstrophy–Imperforate Anus–Spinal Defects) Complex [63]. By ultrasound, a urinary bladder cannot be visualized and an infraumbilical omphalocele with deep umbilical cord insertion and bladder exstrophy are common. The extend of the malformation of the genitalia depend on the extend of bladder exstrophy: in most cases, epispadias or bifid scrotum in male and bifid clitoris in female patients can be detected, but even a cloaca with undetectable genitalia have been described [63, 64].

## Prenatal work-up in the situation of divergence of sonographic and genotypic sex

With the introduction of prenatal genetic testing of the fetus in the setting of fetal abnormalities or maternal age and the introduction of noninvasive prenatal testing (NIPT), a rapidly increasing proportion of fetuses has undergone prenatal testing of its genotypic sex in recent years [65]. Although not considered as primary medical information, nowadays information on fetal sex is consequently readily available to many patients. Discrepancy between NIPT and prenatal ultrasound is reported with an incidence of < 0.01% [66]. Importantly for clinical practice about 1/3 of these cases are related to human or methodologic errors such as mislabeling of blood samples, laboratory methodologic limitations, transcription errors and suboptimal visualization of the fetal external genitalia [66]. It is important to mention that the positive predicted value of NIPT testing for sex chromosomes is significantly lower than for trisomies 21, 18 and 13 [67]. Only ~ 1/3 of cases where sex chromosome aneuploidy is suggested by NIPT are confirmed by pre- or postnatal karyotyping. Consequently in the case of repeated discrepancy between NIPT and prenatal ultrasound, invasive prenatal testing should be offered to the parents [68]. As chorionic villus sampling is associated with an increased frequency of confined placental mosaicism associated with sex chromosome abnormalities, amniocentesis should be the preferred method in this situation [69].

## Prenatal work-up in the situation of ambiguous genitalia

**Fig. 6:**
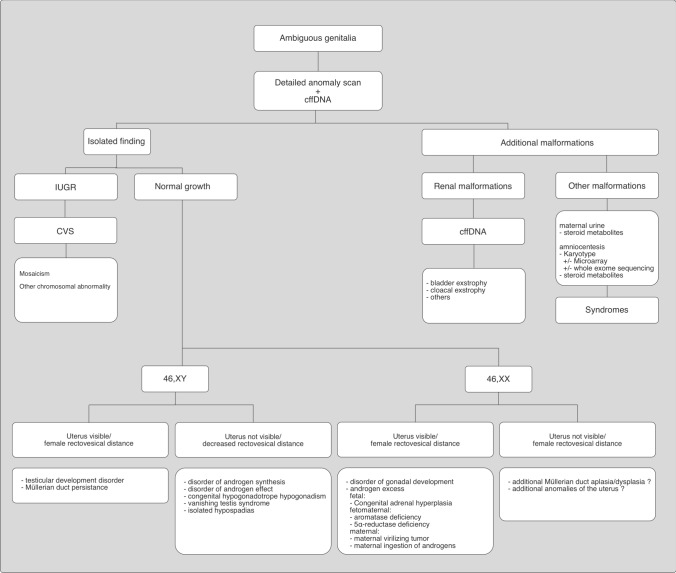
Differential diagnostic procedure in case of sonographic evidence of ambiguous genitalia or divergence between genotypic and phenotypic gender, adapted from [34, 72, 73]

First, the genital anomalies should be described in detail (Fig. 6). Is there a discrepancy between the chromosomal sex and phenotype? Are the ultrasound findings of the external genitalia unclear or conspicuous? Phallus (absent, short, changed shape, epispadia, hypospadia), scrotum (absent or bifid scrotum, cryptorchidism), and female genitalia (virilized female external genitalia with enlarged phallus or fused labia without scrotum yet visible uterus or increased recto-vesical distance) should be described.

Second, a systemic evaluation of the fetal anatomy to identify additional malformations that may guide the diagnosis as delineated above should be performed. Special attention should be laid into the visualization of the fetal internal genitalia and pathognomonic signs of certain syndromes as mentioned above to guide parent counseling. For most cases, 2D-ultrasound will suffice to detect all related abnormalities, yet 3D-ultrasound might aid in the diagnosis in some special cases [32]. Fetal MRI may provide useful information especially in cases with complex genito-urinary malformations.

Third, genetic counseling including a detailed family medical history should be performed. Although simple karyotyping already adds relevant information for the parents, it is insufficient in many conditions. Nowadays, fetal karyotype with FISH for SRY, or fetal karyotype with FISH for SRY and amniotic fluid hormone studies should be offered to the parents [70]. Specific genetic analysis may be performed if the combination of ambiguous genitalia with extragenital malformations suggests a certain syndrome with known genetic cause and in these cases, SNP array and next generation sequencing (exome or whole genome sequencing) will add important information on additional copy number variations (CNV) and mutations, respectively, for parent counseling.

Fourth, prenatal management should include regular sonographic controls to diagnose deterioration of the fetal status. Especially in syndromic fetuses, the development of IUGR and/ or oligohydramnios during the course of pregnancy should be followed. The course of pregnancy or findings during pregnancy might help in the diagnosis. As delineated above, maternal virilization (hirsutism, acne, enlarged clitoris) may indicate an androgen-producing or a placental aromatase defect. Low E3 in the triple test may indicate SLOS, an adrenogenital syndrome, placental aromatase defect, or trisomy 18. A low PAPP-A and PlGF, high HCG and AFP in early pregnancy are indicative of uteroplacental growth restriction.

In the situation of 46,XX DSD, the severity of the 46,XX DSD can be classified according to Prader ranging from simple clitoral hypertrophy (Prader stage I) to the complete fusion of the labioscrotal folds with a phallus-like enlarged clitoris and extension of the urethra (Prader stage V). In the latter, the external genitals resemble the genitals of a boy with absent descent of the testes. Almost always, these changes are the result of early antenatal exposure to high concentrations of androgens. Of those, more than 90% of the cases exhibit a CAH, in more than 90% in the case of 21-hydroxylase deficiency (CYP21A2-CAH). Less commonly, the aforementioned 11β-and 3β-hydroxylase deficiency, fetal androgen production in a bisexual gonad (ovotestis) or androgen-producing Tumor, maternal androgen production (tumor, luteoma), maternal androgen intake (progesterone, danazol) or deficiency of fetoplacental aromatase may lead to fetoplacental hyperandrogenemia. In rare cases, XX DSD with positive SRY a testicular tissue (testicular XX DSD) and Müller derivatives (ovotesticular XX DSD) are present. In case of CAH, the adrenal glands should be imaged by ultrasound, as those might be enlarged. In case of CAH, a prenatal therapy with dexamethasone during the phase of sex differentiation is possible. Because of potential effects of early treatment with dexamethasone on the neurological and neuroendocrinological development of the child, disadvantages of this treatment should be thoroughly discussed with the parents. This should be conducted only in specialized centers, where the current data should be critically reviewed again and again [71]. If treatment is appropriate, the treatment (20 μg/kgKG dexamethasone per day, divided into 3 single doses) starts with 6 weeks of gestation. An early sex determination should be performed. In female fetuses with molecular genetic evidence of CAH, this therapy is continued until birth. In male fetuses and female fetuses without the disease, treatment is discontinued. In girls with very pronounced symptoms of virilization, surgical genital correction might be necessary after birth.

In the situation of XY DSD, the causes are more diverse and remain unexplained in up to 50% of the even postnatally. The testis can be visualized sonographically in some of the disorders. 50–70% of cases are caused by defects of testosterone synthesis or the androgen. Rare cases can be attributed to testicular dysgenesis, a mixed gonadal dysgenesis (X0, XY) and/or are gene mutations on the *SRY*-, *SOX9*- or *WT1* gene.

Last, the need for delivery in a setting of special neonatal care and neonatal surgery should be evaluated. In syndromic forms of DSD, the risk of preterm delivery is increased. Delivery should take place in a hospital with a neonatal care unit that is aware of the special requirements of these children and may organize required postpartum reconstructive surgeries.

With the increase in knowledge on genes and hormones involved in fetal sex differentiation, a thorough prenatal evaluation and parental counseling has been made possible. Still there exist many cases with unknown etiology that warrant further research.
